# The Multiple Platforms Effect (MPE): A quantification of how exposure to similarly biased content on multiple online platforms might impact users

**DOI:** 10.1371/journal.pone.0327209

**Published:** 2025-08-01

**Authors:** Robert Epstein, Amanda Newland, Thomas Peeler, Basil Thaddeus

**Affiliations:** American Institute for Behavioral Research and Technology, Vista, California, United States of America; University of Zurich Irchel Campus: Universitat Zurich Standort Irchel, SWITZERLAND

## Abstract

Over the past decade, controlled studies have identified nearly a dozen new forms of manipulation that can be used by search engines, social media platforms, microblogging platforms, and intelligent personal assistants. A recent study has shown that when users were exposed repeatedly to similarly biased content on the same platform, the net impact of those exposures was additive. We now ask the following: What happens when users are exposed to similarly biased content generated by different means on multiple online platforms? In the present experiment, which was randomized, controlled, counterbalanced, and double-blind, we exposed people to similarly biased content generated by different means on three different platforms – simulations of Google Search, Alexa, and X (f.k.a., Twitter) – presented successively and in a random order. We found that the impact of successive exposures was additive for both opinions and voting preferences pertaining to political candidates. Overall, the number of undecided voters voting for the favored candidate increased with each successive platform exposure by 42.4%, then 56.5%, then 66.7% over the pre-exposure level. We speculate that if Big Tech companies share values or political preferences, their net effect on our elections might be considerably greater than the effect of any individual company.

## 1. Introduction

In recent years, a number of controlled studies have been published that demonstrate and quantify the impact of new forms of influence that the internet has made possible, among them the search engine manipulation effect (SEME) [1, cf. 2–8], the search suggestion effect (SSE) [9], the answer bot effect (ABE) [10], and the targeted messaging effect (TME) [11]. A new study on the multiple exposure effect (MEE) has also demonstrated that when users were exposed to similarly biased content repeatedly on the same platform (at least for simulations of the Google search platform, the X [f.k.a. Twitter] platform, and the Alexa intelligent personal assistant [IPA]), the combined impact of those exposures was additive [5].

The additive impact was not surprising when viewed in the context of a large number of studies that have been conducted for over a century on the impact of repeated stimulus presentations [e.g., 12–14]. Although there are cases in which repeated stimulus presentations may weaken responding [e.g., 15–18], it is more common for repeated stimulus presentations to strengthen it [e.g., 19–28]. In the case of MEE, the additivity was measured by looking at the post-manipulation percentage increase in the number of participants voting for the political candidate favored in the manipulation. Beginning with a group of undecided voters who initially were split 50/50 in their support for one of two political candidates, they were randomly assigned either to a pro-Candidate-A group or a pro-Candidate-B group. Then they were allowed to conduct research on a platform in which the political bias of the content favored Candidate A or B (the manipulation), after which their voting preferences were measured again. The percentage increase in the number of participants now voting for the favored candidate was between 14% and 70%, depending on the platform [5, cf. 1,10, 11]. After a second research period in which the same group of participants was exposed to similarly biased content on the same platform, that percentage increased further, to between 20% and 90% [5]. And after a third research period, that percentage increased still further, to between 22% and 98% [5].

The results of the MEE study raise an obvious question: What if online users – ideally, people who are undecided on some issue and who therefore might be vulnerable to online influence that might help them make up their minds – were exposed to similarly biased content generated by entirely different mechanisms on different online platforms? The answer to this question is by no means obvious, because similarly biased content – say content that favors one political candidate – might be presented in very different forms on different platforms. On a search engine, it might come in the form of high ranking search results that link to webpages that favor one candidate. On a website showing newsfeeds, it might come in the form of high ranking links to news stories with that leaning. On a video website such as YouTube, it might come in the form of an “up-next” or a recommended video that is biased in that way. With content coming in so many different forms, there is no reason to believe a priori that the impact of multiple exposures will be additive.

We hope we are not overstating the issue, but we believe that having gotten clear findings in the MEE study [5], it would have been intellectually dishonest of us to have asserted, without evidence, that exposure to similarly biased content on different platforms would necessarily have produced additive effects.

In the present study, we conducted a single randomized, controlled, counterbalanced, and double-blind experiment to measure the impact of presenting similarly biased content to users on simulations of three popular online platforms: Google search, X, and the Alexa IPA.

## 2. Methods

### 2.1 Ethics statement

The federally registered Institutional Review Board (IRB) of the sponsoring institution (American Institute for Behavioral Research and Technology) approved this study with exempt status under HHS rules because (a) the anonymity of participants was preserved and (b) the risk to participants was minimal. The IRB is registered with OHRP under number IRB00009303, and the Federalwide Assurance number for the IRB is FWA00021545. Informed written consent was obtained as specified in the Procedure section below.

### 2.2 Participants

Participants were recruited online from the Amazon Mechanical Turk (MTurk) subject pool between May 28th and June 12th, 2024. Participants were screened by CloudResearch to prevent bots and suspect users from entering our subject pool. Participants were required to be at least 18 years old, and they were allowed to participate only if they answered Yes to the question, “Are you eligible to vote in the United States?” and No to the question, “Do you know a lot about politics in Australia?” We also asked participants to rank how familiar they were with both Bill Shorten and Scott Morrison – two men who ran for Prime Minister of Australia in 2019 – on a scale from 1 to 10, where 1 was labeled “Not at all” and 10 was labeled “Quite familiar”; we eliminated participants who answered above a 3 on either scale. We screened our participants in these ways to try and ensure that they would be “undecided” about whom they might vote for [cf. 1]. After screening, we had data from 536 participants to analyze.

The participants ranged in age from 18 to 82 (*M* = 37.9, median = 36, *SD* = 11.1). 62.1% (*n* = 333) of the participants identified themselves as female, 36.4% (*n* = 195) as male, and 1.5% (*n* = 8) chose not to identify their gender. 75.0% (*n* = 402) of the participants identified themselves as White, 10.8% (*n* = 58) as Black, 5.6% (*n* = 30) as mixed, 4.9% (*n* = 26) as Asian, and 3.7% (*n* = 20) as other. Regarding the level of education people reported having completed, 0.9% (*n* = 5) said none, 5.6% (*n* = 30) said primary, 33.2% (*n* = 178) said secondary, 44.2% (*n* = 227) said bachelor’s degree, 15.1% (*n* = 81) said master’s degree, and 2.8% (*n* = 15) said doctorate. 39.6% (*n* = 212) of the participants identified themselves as liberal, 30.8% (*n* = 165) as moderate, 18.8% (*n* = 101) as conservative, 7.6% (*n* = 41) as none, and 3.2% (*n* = 17) as other. The mean familiarity score for Scott Morrison was 1.05 (*SD* = 0.27), and the mean familiarity score for Bill Shorten was 1.02 (*SD* = 0.16).

### 2.3 Procedure

Participants were given brief instructions and were asked for their consent to participate in the study (S1 Text). They were then asked a series of basic demographic questions, after which they were given brief, roughly equivalent, descriptions of the two political candidates (S2 Text). Then we asked them eight questions about the candidates – three opinion questions about each candidate (regarding liking, trusting, and overall impression) (S1 Fig) and two questions about their voting preferences. In the first, participants indicated their voting preference on an 11-point scale from 5 to 0 to 5, with the names of the candidates counterbalanced at each end. Finally, they were asked a forced-choice question: “If you had to vote right now, who would you vote for?”.

They were then assigned at random to either Group 1 (pro-Morrison), Group 2 (pro-Shorten), or a control group; this was done without the participant’s knowledge. At this point, one of the three platforms (mentioned above) loaded into place for each participant, and he or she was given brief instructions on how to use that platform to conduct further research on the candidates (S2 Fig).

We will now briefly describe the manipulation performed on each platform; bear in mind, however, that the order of platform presentation was randomized for each participant. The order in which we describe them here is not necessarily the order in which each participant saw them. (We belabor this point in order to avoid subjecting our readers to an order effect).

**Fig 1:**
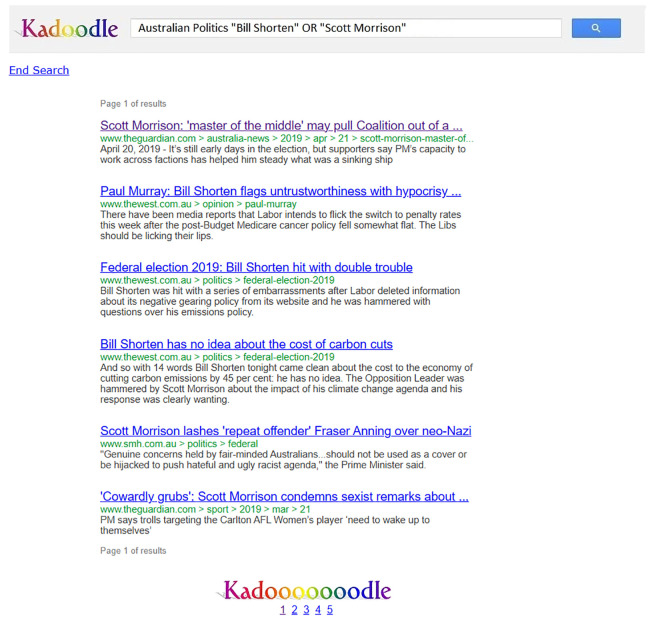
Example of Kadoodle search results page. Reprinted from [3] under a CC BY license, with permission from the American Institute from Behavioral Research and Technology, original copyright 2024. This figure is similar but not identical to the original image and is therefore for illustrative purposes only.

On Kadoodle, our simulation of Google search, participants were instructed to conduct research on the two candidates by clicking on one or more search results and then reading the content on the pages linked to those search results (S3 Text). Each participant had access to five pages of search results, with six results per page (Fig 1). They could switch between pages by clicking on a link at the bottom of each page, just as one can on Google search (Fig 1).

**Fig 2:**
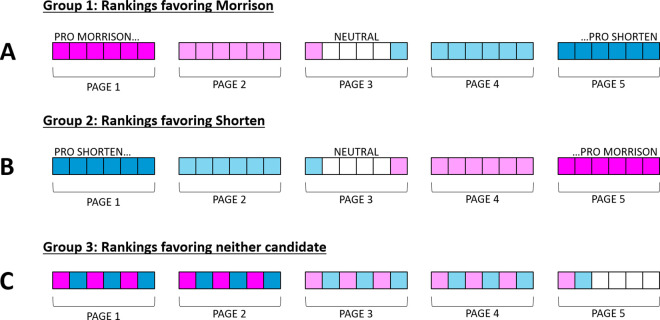
Position and content of Kadoodle search results for each bias group. Participants in Group 1 saw search results favoring Scott Morrison (dark pink indicates stronger bias). Participants in Group 2 saw search results favoring Bill Shorten (dark blue indicates stronger bias). Participants in Group 3 saw no bias in search results.

All the search results were real, sourced from Google.com. The webpages were real, as well, although we removed all active links from each page. The only difference between what the three different groups saw in this experiment was in the ordering of the search results. In Group 1, the ordering favored Scott Morrison (in other words, the higher the search result in the list, the more the content in the linked webpage favored Morrison); in Group 2, the ordering favored Bill Shorten; and in Group 3, the ordering was mixed, favoring neither candidate (Fig 2). The content in the webpages favored one particular candidate by showing positive information about that candidate, negative information about his opponent, or both. The ranking of the webpages was based on bias ratings provided by five independent observers.

After participants clicked on at least one search result, a button appeared in the upper-left part of the screen that allowed participants to end the search. If a full 5 minutes passed, participants were notified that the search period was over. When the search ended, participants were again asked those eight questions we described earlier (six opinion questions, and two voting-preference questions). After these questions were answered, participants were informed that they could now conduct further research about the candidates on another platform.

**Fig 3:**
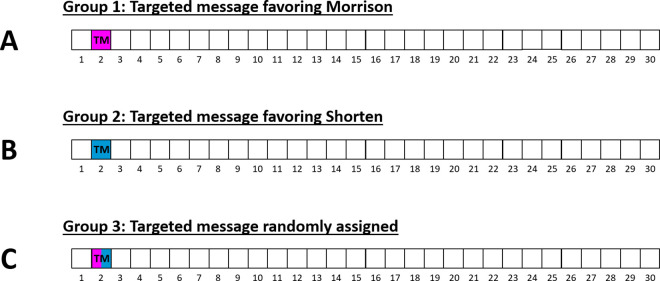
Position and content of Twiddler alert for each bias group. In Group 1, users saw one targeted message in the second position which was biased toward Scott Morrison (dark pink). In Group 2, users saw one targeted message in the second position which was biased toward Bill Shorten (dark blue). In Group 3, users saw one targeted message which was randomly chosen to be either pro-Morrison or pro-Shorten.

**Fig 4:**
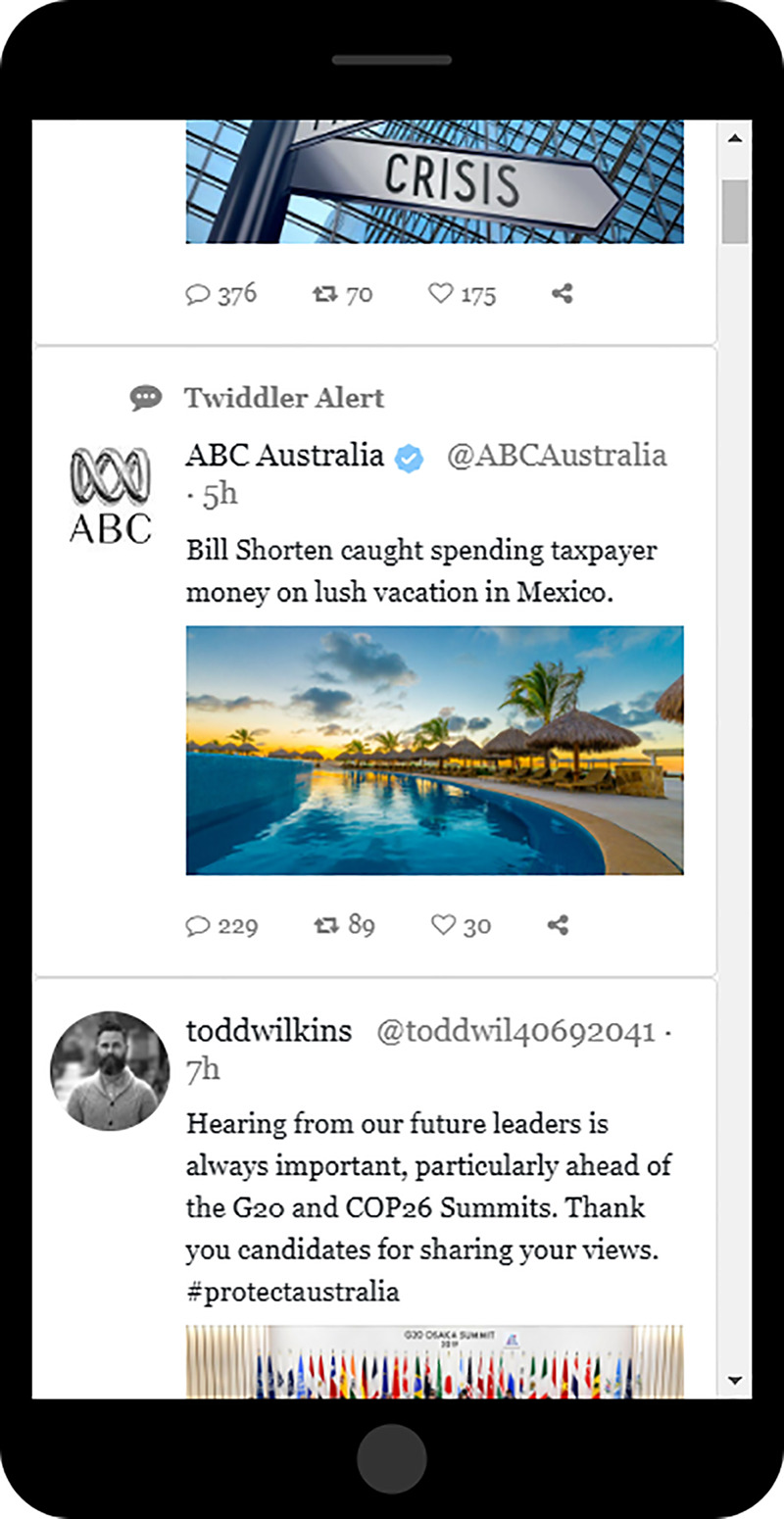
Example of Twiddler content. Reprinted from [11] under a CC BY license, with permission from the American Institute from Behavioral Research and Technology, original copyright 2023. This figure is similar but not identical to the original image and is therefore for illustrative purposes only.

On Twiddler, our X simulation, participants were told to scroll from the beginning to the end of a list of 30 tweets (S4 Text). They were also told, “Your task is to use this Twiddler feed to try to further clarify your views on each candidate so that you are better able to decide which one deserves your vote.” 29 of the tweets in the feed of 30 appeared to come from regular users and were neutral in their content, meaning that they were not biased to favor either of the candidates. These 29 tweets were the same for all participants. The manipulation involved only the special tweet in the 2nd position (Fig 3). That tweet was apparently sent by the company (rather than by a user), and it was labeled, “Twiddler alert.” It contained a brief description of a negative news story about the opponent to the candidate favored in all the manipulations seen by that participant. The same negative news story was seen by every participant in the two bias groups. The only difference was the name at the beginning of the headline. So if the content in each manipulation always favored Bill Shorten, the news alert read, “Scott Morrison caught spending taxpayer money on lush vacation in Mexico.” If Scott Morrison was the favored candidate, the news alert read “Bill Shorten caught spending taxpayer money on lush vacation in Mexico.” Participants could move on to the next page by clicking the “Continue” button below and to the left of the phone image (Fig 4). Participants were then presented with those eight questions again, and then given brief instructions for how to continue their research on a third platform.

**Fig 5:**
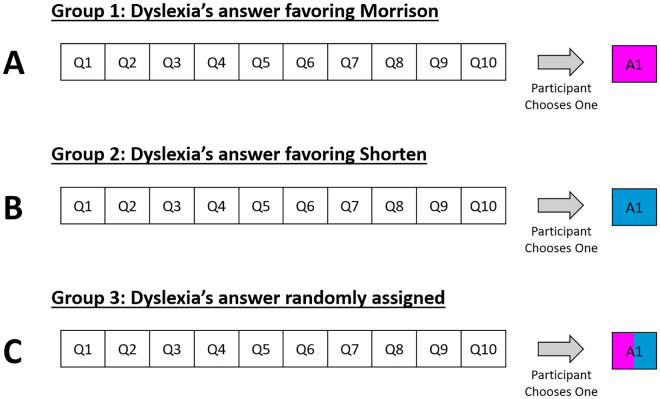
Dyslexa questions and answers for each bias group. In Group 1, participants chose one question out of the 10, and the answer was biased toward Scott Morrison (dark pink). In Group 2, participants chose one question, and the answer was biased toward Bill Shorten (dark blue). In Group 3, participants chose one question, and the answer was randomly chosen to be either pro-Scott or pro-Morrison.

**Fig 6:**
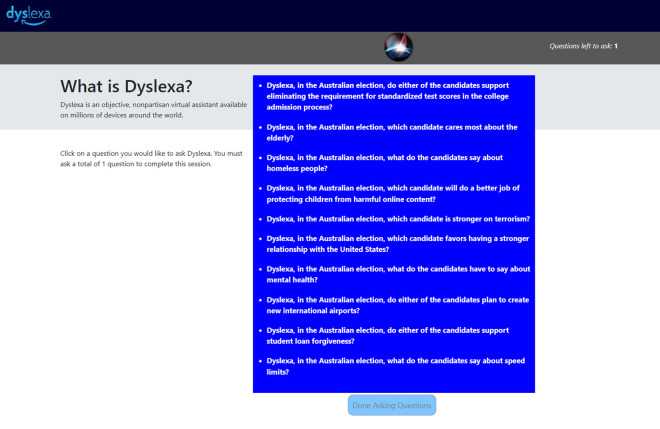
Example of Dyslexa content. Reprinted from [10] under a CC BY license, with permission from the American Institute from Behavioral Research and Technology, original copyright 2022. This figure is similar but not identical to the original image and is therefore for illustrative purposes only.

On Dyslexa, our Alexa simulator, participants were told how to use this IPA (S5 Text), and they were then shown a list of 10 questions that were phrased to ask Dyslexa which candidate had stronger policies on 10 separate political issues (see Fig 5 and S6 Text). They could ask one question by clicking it, after which Dyslexa, using the original Alexa voice (Amazon Polly), responded orally with an answer supportive of Morrison, Shorten, or, in the control group, either candidate (depending on which of these three groups the participant had been assigned to at the beginning of the experiment). After Dyslexa finished answering the question, the remaining questions would be greyed out, and participants could proceed by clicking the “Done Asking Questions” button that appeared directly below the list of 10 questions (Fig 6). When research on the third platform was completed, participants were again asked those eight questions – six opinion questions and two voting-preference questions.

Finally, participants were asked whether anything “bothered” them about the content that had been displayed during the searches. We asked this question to try to determine whether the participants in the two bias groups perceived any bias in the content. We could not ask directly about the perception of bias, because leading questions of this sort have long been known to inflate answers artificially [29].

## 3. Results

Our metric of choice for measuring the effect of platform manipulation on votes was Vote Manipulation Power (VMP), defined as the percentage increase in the number of votes for a given candidate as a result of a manipulation in favor of that candidate (S7 Text). Because the experiment employs random assignment, we combined the two bias groups in this computation.

In the bias groups, VMP increased with each successive exposure to similarly biased content on different platforms, with the most dramatic change seen after the first exposure (Table 1). Differences between VMPs after exposure to content on each successive platform were also found to be significant in pairwise comparisons (Table 2). Significant differences between VMPs for exposure to content on each successive platform were also found for demographic groups: gender, age (after second platform only), level of education, race/ethnicity, and political affiliation (S1–S5 Tables). An especially large difference was found for susceptibility to this manipulation by political leaning. Liberals were highly susceptible, achieving a VMP of 62.0% after exposure to content on the third platform; whereas, conservatives had a VMP of only 35.3% at this point.

**Table 1:** Effects of biased content on voting preferences (VMP), bias groups combined, n = 365.

Platform	*n*	VMP (%)	McNemar’s Test $X^{2}$	*p*
**1**	365	42.4	46.02	<.001
**2**	–	56.5	72.07	<.001
**3**	–	66.7	92.49	<.001

**Table 2:** Pairwise comparisons of VMP for each platform use.

	*n*	VMP (%)
**Platform 1**	365	42.4
**Platform 2**	365	56.5
**Difference**	–	14.1
**Statistic**	–	*z* = − 3.81
***p***	–	<.001
**Platform 2**	365	56.5
**Platform 3**	365	66.7
**Difference**	–	10.2
**Statistic**	–	*z* = − 2.83
***p***	–	=.004
**Platform 1**	365	42.4
**Platform 3**	365	66.7
**Difference**	–	24.3
**Statistic**	–	*z* = − 6.59
***p***	–	<.001

Regarding scores on the 11-point voting preferences scale, prior to exposure to content on any of the platforms, we found no significant differences in mean scores by group (Pro-Scott Morrison, Pro-Bill Shorten, and Control) (S6 Table). In the two bias groups (Groups 1 and 2), significant differences emerged between the pre-manipulation scores and the post-manipulation scores on this scale after each successive exposure to similarly biased content on different platforms (Table 3). In the control group, there was no significant difference between pre-exposure voting preferences on the 11-point scale and voting preferences on this scale after each successive platform use (S7 Table).

**Table 3:** Changes in voting preferences for the favored candidate measured on an 11-point scale, two bias groups combined (corrected such that a positive value indicates preference for the favored candidate).

	Pre-Exposure Likely Vote for Favored Candidate, Mean (SD)	Post-Exposure Likely Vote for Favored Candidate, Mean (SD)	Mean Difference^†^	*z* ^‡^	*p*
**Platform 1**	−0.21 (2.70)	1.20 (2.74)	1.40	−9.06	<.001
**Platform 2**	–	1.71 (2.70)	1.91	−10.84	<.001
**Platform 3**	–	2.07 (2.65)	2.27	−11.77	<.001

*Note*: The means from 2nd platform and 3rd platform are being compared to the pre-exposure mean.

† The absolute value of the mean difference is shown.

‡ The z values come from a Wilcoxon signed ranks test between post-exposure and pre-exposure ratings for the favored candidate.

Regarding the six opinion questions we asked, prior to any manipulations, we found no significant differences between any of the bias or control groups in these ratings (S8 Table). In the control group, regarding the differences between pre-exposure ratings and post-exposure ratings, no significant difference was found between the two candidates for any successive platform use (S9 Table). In the two bias groups combined, mean differences in ratings after successive exposures to content on each of the platforms were minimal for the favored candidate, but showed consistent downward trends for the non-favored candidate, so the net differences in opinions were highly significant (Table 4). The large negative shifts in opinions for the non-favored candidate may be due to negativity bias [9,30,31]. In other words, our participants might be paying more attention to negative language about the non-favored candidate than they do to positive language about the favored candidate.

**Table 4:** Pre- and post-exposure opinion ratings of the favored and non-favored candidate measured on a 10-point scale, bias groups only.

		Favored Candidate Mean (SD)			Non-Favored Candidate Mean (SD)			
		Pre	Post	Diff	Pre	Post	Diff	*z* ^†^
**Platform 1**	**Impression**	7.10 (1.88)	6.94 (2.19)	− 0.16	7.16 (1.81)	5.39 (2.22)	− 1.76	−9.45^***^
	**Likeability**	6.95 (1.94)	6.79 (2.20)	− 0.16	7.05 (1.85)	5.41 (2.19)	− 1.64	−9.04^***^
	**Trust**	5.96 (2.01)	6.16 (2.28)	+ 0.19	6.09 (2.00)	4.93 (2.26)	− 1.16	−8.60^***^
**Platform 2**	**Impression**	–	7.02 (2.16)	− 0.08	–	4.74 (2.12)	− 2.42	−11.62^***^
	**Likeability**	–	6.82 (2.22)	− 0.13	–	4.69 (2.22)	− 2.36	−11.24^***^
	**Trust**	–	6.33 (2.39)	+ 0.36	–	4.33 (2.21)	− 1.76	−10.80^***^
**Platform 3**	**Impression**	–	7.07 (2.27)	− 0.04	–	4.32 (2.12)	− 2.84	−12.45^***^
	**Likeability**	–	6.89 (2.33)	− 0.06	–	4.24 (2.21)	− 2.81	−12.21^***^
	**Trust**	–	6.43 (2.43)	+ 0.47	–	4.00 (2.20)	− 2.09	−11.84^***^

*Note*: The means from 2nd platform and 3rd platform are being compared to the pre-exposure mean.

† The z values come from Wilcoxon signed ranks test between post-exposure minus pre-exposure ratings for the favored candidate and the post-exposure minus pre-exposure ratings for the non-favored. candidate.

*** **p* *< .001.

Regarding the search behavior of our participants on Kadoodle, our participants were less likely to click on low-ranking search results, and the majority of clicks were on the first page of search results (S3 Fig). We also found that participants spent more time on webpages that corresponded to highly ranked search results (S4 Fig). This pattern of clicks and time spent on webpages was found in both bias groups and the control group. We found these same patterns whether the search engine was presented in the 1st, 2nd, or 3rd positions. These patterns of clicks and time spent on webpages were similar to patterns we have found in many other experiments we have conducted on our search engine simulator [1,4,5].

Regarding perception of bias in content, only 5.48% (*n* = 20) of participants in the two bias groups showed awareness of bias on any of the platforms. This is far below what has been observed previously in experiments with a biased search engine, and falls closer to the bias detection rate when participants are exposed to targeted messaging on social media [1,11]. Presumably, this is because the search engine was one of only three platforms participants saw.

Contrary to what common sense might suggest, previous research has generally shown people who show awareness of bias in online content shift their views even farther in the direction of that bias than people who seem unaware of the bias [1; cf. 10,32]. This occurs presumably because people tend to trust algorithmic output as being inherently impartial or objective [2, 33–36, cf. 37]. In the present experiment, awareness of bias once again appeared to increase susceptibility to bias. Specifically, the VMP for participants who correctly identified bias was found to be significantly greater than the VMP for those who did not, with the difference becoming greater with each successive platform use (First platform: VMP_*unnoticed*_ = 41.4%, VMP_*noticed*_ = 62.5%, *z* = 1.86, *p* = .031; Second platform: VMP_*unnoticed*_ = 55.0%, VMP_*noticed*_ = 87.5%, *z* = 2.85, *p* = .002; Third platform: VMP_*unnoticed*_ = 65.1%, VMP_*noticed*_ = 100%, *z* = 3.23, *p* < .001).

## 4. Discussion

### 4.1 Summary and conclusions

This study is the 13th in a series of studies that have been published or that are currently under review; these studies measure the impact of new forms of influence made possible by the internet. The series began with a report about the “search engine manipulation effect” (SEME) which was published in the *Proceedings of the National Academy of Sciences* in 2015 [1]. That study, which was based on data from five randomized, controlled experiments with 4,556 participants from the US and India, showed that bias in search results can easily shift the voting preferences of between 20% and 80% of undecided voters without their knowledge. That study has been partially or fully replicated multiple times [2–8]. In another SEME study, we showed that bias in search results can – for people who have not yet made up their minds – shift people’s opinions about non-political topics – perhaps, we speculated, about any topic at all [3]. In another SEME study, we presented evidence suggesting that SEME is an unusually large list effect because it is maintained by a daily regimen of operant conditioning. Because most searches are for simple facts (“What is the capital of Kentucky?”), and because correct answers invariably occur in the highest-ranking search result, people learn to trust high-ranking search results over lower ones. When, at some point, someone asks an open-ended question (“What is the best guitar brand?”), people continue to trust, and hence to click, high-ranking results [4].

Our most recent studies, published or submitted since 2023, have reported findings on the targeted messaging effect (TME) [11], the search suggestion effect (SSE) [9], the video manipulation effect (VME) [32], the opinion matching effect (OME) [38], the digital personalization effect (DPE) [39], and the multiple exposure effect (MEE) [5]. Using simulations of three different popular online platforms – Google search, X, and Alexa – the MEE study showed that when people were exposed repeatedly to similarly biased content on the same platform, the impact of the manipulation increased with each exposure; in other words, MEE appears to be an additive effect.

The present study examines the impact of exposure to similarly biased content generated by different means on simulations of multiple online platforms – once again, Google search, X, and Alexa. We gave participants access to three different platforms, with those platforms presented in a random order. The results were similar to those we found in the MEE study: The number of undecided voters voting for the favored candidate increased with each successive platform exposure by 42.4%, then 56.5%, and then 66.7% over the pre-exposure level of voting preference; each of the three increments was statistically significant.

If tech platforms were like news organizations – with thousands of such organizations, each with its own viewpoint, competing for our attention every day – the increments we found in our experiment would be relatively harmless. The problem we all face is that the internet is currently dominated by a small number of large monopolies [see 40] that share similar cultural and political values [41]. That overlap in values could change, of course, but our MPE findings remind us about a possible downside to homogeneity on the internet. Repeated exposures to similarly biased content generated by different mechanisms on different platforms might be impacting people additively. The overlap in bias might occur because of shared values at large companies, but it might also occur when people get trapped in filter bubbles [42, cf. 43], where they are being shifted from one small platform to another, each platform promoting similar values or political viewpoints.

The bottom line here, which we see as a matter of some concern, is that platforms that seem to serve very different purposes (say, TikTok versus Facebook) might generate content that is similarly biased. When this occurs, the impact on users might be additive.

### 4.2 Limitations and future research

Our study suffers from a number of limitations, some of which we are attempting to understand and remedy in ongoing research. The most obvious limitations result from our procedure. We are presenting three different platforms one right after the other; how, if at all, would our results change if we spaced these presentations out over time? In the real world, people might sometimes move quickly from one platform to another and, in so doing, might be exposed to similarly biased content. With spaced exposures – depending on the magnitude of the time intervals between exposures – one could argue that the impact might be larger or smaller than the impact of immediately consecutive exposures [44]. We also presented only three platforms; would the impact of more exposures produce diminishing returns? We also did not attempt to measure how long the impact of our manipulations would last. If you are trying to influence voters, that is an important issue.

We also did not examine the possible impact of inconsistent content. What if two platforms presented content favoring Candidate A and a third platform presented content favoring Candidate B? Might inconsistent content produce only small or no effects, just as content in control groups often does [45]? Bear in mind that a single platform has a high degree of control over the content it presents to its users; whereas, content impacting users on different platforms might vary in ways that undermines the combined effects of such content.

As always, we worry about ways in which participants we obtain from MTurk might differ from the population of eligible voters in the US [46, 47]. On this issue, we can note only that we have used a third party quality assurance company (CloudResearch) to screen out bots and suspicious users, and that we ended up with a diverse sample of 536 people who claimed to be registered US voters (see S1–S5 Tables). Given the pattern and size of the effect we found, we have no reason to believe that it occurred because of defects in our sample. We acknowledge that because we only had US voters in our sample, further research should be conducted to determine whether our results are applicable to voters in other countries.

Perhaps more important is the fact that our design selected for a particular type of participant. The sample was composed not only of undecided voters – an important criteria for inclusion in most of our experiments – but almost exclusively of a particular *type* of undecided voter – namely, the “low-information” voter. Such voters are known to be different in some respects from high-information voters [48], but, again, we have no reason to believe that conducting our experiment with low-information voters casts doubt on our findings.

We note also that we only examined MPE in the present study using simulations of three platforms. Additional research is needed to determine the extent to which our findings are applicable to platforms such as YouTube, Facebook, Instagram, TikTok, and so on. We recently completed the building of a simulated Facebook environment so that we can begin to assess how various kinds of content might be affecting Facebook users.

Finally, we note that we detected post-manipulation changes in our participants by asking eight questions that measured opinions and voting preferences. How might other political behavior be influenced? In particular, to what extent might the shifts we found in voting preferences be predictive of shifts in actual votes? That, too, is a matter to be explored in future research.

## Supporting information

S1 Text Informed consent statement. (DOCX)

S2 Text Candidate biographies. (DOCX)

S3 Text Instructions immediately preceding Kadoodle simulation. (DOCX)

S4 Text Instructions immediately preceding Twiddler simulation. (DOCX)

S5 Text Instructions immediately preceding Dyslexa simulation. (DOCX)

S6 Text Alexa simulator, “Dyslexa,” questions and answers. (DOCX)

S7 Text Vote Manipulation Power (VMP). (DOCX)

S1 Fig Opinion and voting questions. (DOCX)

S2 Fig MPE procedure. (DOCX)

S3 Fig Average number of clicks per search result position by exposure number. (DOCX)

S4 Fig Average time spent per search result position by exposure number. (DOCX)

S1 Table Demographic analysis by age. (DOCX)

S2 Table Demographic analysis by gender. (DOCX)

S3 Table Demographic analysis by education level. (DOCX)

S4 Table Demographic analysis by race/ethnicity. (DOCX)

S5 Table Demographic analysis by political affiliation. (DOCX)

S6 Table Pre-exposure voting preferences measured on an 11-point scale, split by bias group (such that a negative value indicates preference for Scott Morrison and a positive value indicates preference for Bill Shorten). (DOCX)

S7 Table Changes in voting preferences measured on an 11-point scale, control group only (such that a negative value indicates preference for Scott Morrison and a positive value indicates preference for Bill Shorten). (DOCX)

S8 Table Pre-exposure opinion ratings of Bill Shorten and Scott Morrison measured on a 10-point scale, split by bias group. (DOCX)

S9 Table Pre- and post-exposure opinion ratings of Scott Morrison and Bill Shorten measured on a 10-point scale, control group only. (DOCX)
