# Supplementary material for: The Multiple Platforms Effect (MPE): A quantification of how exposure to similarly biased content on multiple online platforms might impact users
Source: PLoS One. 2025 Aug 1;20(8):e0327209. doi: 10.1371/journal.pone.0327209 (PMC12316238; doi:10.1371/journal.pone.0327209)
Supplement: S1 Text — (DOCX) [file pone.0327209.s001.docx]

**S1 Text. Informed consent statement.**

To participate in this study you must check the box below to give your consent to the following:

I am 18 years or older and I understand that my participation is voluntary, that I am free to withdraw at any time, that I am providing information anonymously and that demographic information collected is confidential and cannot be used to identify me. I agree to allow the data collected to be used for future research projects, and I understand that completion and submission of this survey implies my consent to participate in the present study.
