# Supplementary material for: The Multiple Platforms Effect (MPE): A quantification of how exposure to similarly biased content on multiple online platforms might impact users
Source: PLoS One. 2025 Aug 1;20(8):e0327209. doi: 10.1371/journal.pone.0327209 (PMC12316238; doi:10.1371/journal.pone.0327209)
Supplement: S2 Text — (DOCX) [file pone.0327209.s002.docx]

**S2 Text. Candidate biographies.**

**Scott Morrison** was born in Waverley, New South Wales (AUS) on May 13th, 1968. He completed a Bachelor of Science honors degree in applied economic geography at the University of New South Wales. Morrison married his high school sweetheart, Jenny Warren, in 1990 and has two daughters. After graduating from the University of New South Wales, Morrison worked as a national policy and research manager for the Property Council of Australia before moving to New Zealand in 1998 to become the director of the Office of Tourism and Sport. He left this position a year before the contract schedule and returned to Australia in 2000. In 2004, he became the inaugural managing director of Tourism Australia until July 2006.

**Bill Shorten** was born in Fitzroy, Victoria (AUS) on May 12th, 1967. While Shorten was studying at Monash University, he was an active student in the university’s politics club. In 1986, Shorten helped establish a group called Network and briefly served as a private in the Australian Army Reserve from 1985 to 1986. After graduating Monash University with a Bachelors of Arts in 1989 and a Bachelors of Law in 1992, Shorten worked as a lawyer for Maurice Blackburn Cashman for twenty months. In 1994, he worked as a trainee organizer and later accepted a position as a politics national secretary in 2001 and again in 2005. Shorten is currently married to Chloe Bryce and has a daughter.
