# Supplementary material for: The Multiple Platforms Effect (MPE): A quantification of how exposure to similarly biased content on multiple online platforms might impact users
Source: PLoS One. 2025 Aug 1;20(8):e0327209. doi: 10.1371/journal.pone.0327209 (PMC12316238; doi:10.1371/journal.pone.0327209)
Supplement: S3 Text — (DOCX) [file pone.0327209.s003.docx]

**S3 Text. Instructions immediately preceding Kadoodle simulation.**

Participant Instructions:

You will now be given the opportunity to conduct some research on the previously mentioned candidates using Kadoodle. Your goal is to try to clarify your views on each candidate so that you are better able to decide which one deserves your vote.

Use the search engine results we show you as you would normally use any search engine results, and please do not use other search engines to help you. That will invalidate your participation in our study. If you would like to conduct further research on the candidates after you complete our study, go right ahead, but please complete our study first!

You will have a total of 5 minutes to conduct your search. The program will automatically let you know when the time is up.

Please do NOT close the window after conducting your search. Doing so will make it impossible for you to complete our study. Instead, if you feel you have enough information to make a clear choice between the candidates, you may end your search early by clicking "End Search" in the upper left corner of the results page.

PLEASE NOTE: Some web pages might take a while to load, so please be patient.

Click below to continue.
