# Supplementary material for: The Multiple Platforms Effect (MPE): A quantification of how exposure to similarly biased content on multiple online platforms might impact users
Source: PLoS One. 2025 Aug 1;20(8):e0327209. doi: 10.1371/journal.pone.0327209 (PMC12316238; doi:10.1371/journal.pone.0327209)
Supplement: S4 Text — (DOCX) [file pone.0327209.s004.docx]

**S4 Text. Instructions immediately preceding Twiddler simulation.**

Participant Instructions:

On the next page, you will now be shown a phone screen that will allow you to search through a Twiddler feed showing comments people have made about protecting Australia. That feed will include information on the two candidates - Scott Morrison and Bill Shorten.

Your task is to use this Twiddler feed to try to further clarify your views on each candidate so that you are better able to decide which one deserves your vote.

Please scroll through the entire Twiddler feed before making up your mind. Thanks!

Click the 'Continue' button below.
