# Supplementary material for: The Multiple Platforms Effect (MPE): A quantification of how exposure to similarly biased content on multiple online platforms might impact users
Source: PLoS One. 2025 Aug 1;20(8):e0327209. doi: 10.1371/journal.pone.0327209 (PMC12316238; doi:10.1371/journal.pone.0327209)
Supplement: S5 Text — (DOCX) [file pone.0327209.s005.docx]

**S5 Text. Instructions immediately preceding Dyslexa simulation.**

Participant Instructions:

You will now be given the opportunity to ask the Dyslexa virtual assistant 1 question about the candidates from a list of 10 different questions we'll show you.

Your task is to ask Dyslexa questions that will help you further clarify your views on each candidate so that you are better able to decide which one deserves your vote.

To make sure you hear Dyslexa's answer, **PLEASE MAKE SURE YOUR SOUND IS ON!** You will NOT be able to hear her if your sound is off.

Click the 'Continue' button below.
