# Supplementary material for: The Multiple Platforms Effect (MPE): A quantification of how exposure to similarly biased content on multiple online platforms might impact users
Source: PLoS One. 2025 Aug 1;20(8):e0327209. doi: 10.1371/journal.pone.0327209 (PMC12316238; doi:10.1371/journal.pone.0327209)
Supplement: S7 Text — (DOCX) [file pone.0327209.s007.docx]

**S7 Text. Vote Manipulation Power (VMP).**

Vote Manipulation Power (VMP) is calculated as follows:

$$\left( \frac{p'-p}{p} \right)\times100$$

where *p* is the total number of people who voted for the favored candidate pre- manipulation, and *p'* is the total number of people who voted for the favored candidate post-manipulation. If, pre-manipulation, a group of 100 people is split 50/50 in the votes they give us, and if, post-manipulation, a total of 67 people now vote for the favored candidate, the VMP is:

$$\left( \frac{67-50}{50} \right)\times100$$

or 34%. Because *p'* is 17 points larger than *p*, the win margin is 34 (2 × 17, or 34%), and the final vote is 67 to 33, with the favored candidate the winner. So in any group in which the vote is split 50/50 pre-manipulation, the VMP is also the win margin. Note that 17 individuals did not need to *shift* to produce this win margin. We only needed the *net* number of people voting for the favored candidate to be 67. As a practical matter, that net is the key statistic a campaign staff would likely want to know.
