# Supplementary figures and images for: The Multiple Platforms Effect (MPE): A quantification of how exposure to similarly biased content on multiple online platforms might impact users
Source: PLoS One. 2025 Aug 1;20(8):e0327209. doi: 10.1371/journal.pone.0327209 (PMC12316238; doi:10.1371/journal.pone.0327209)

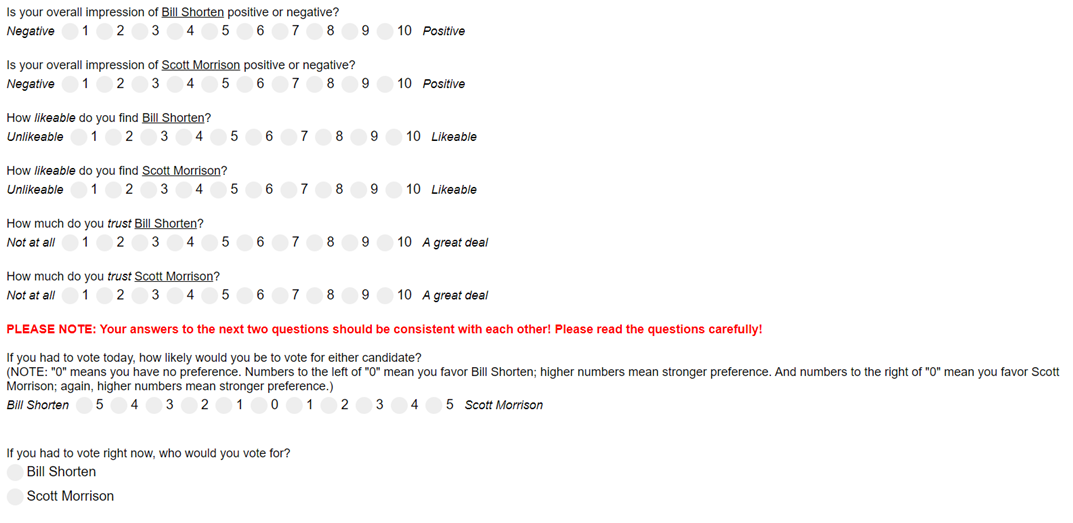


**S1 Fig. Opinion and voting questions.**

Supplement: S1 Fig — (DOCX) [file pone.0327209.s008.docx]
