# Supplementary material for: The Multiple Platforms Effect (MPE): A quantification of how exposure to similarly biased content on multiple online platforms might impact users
Source: PLoS One. 2025 Aug 1;20(8):e0327209. doi: 10.1371/journal.pone.0327209 (PMC12316238; doi:10.1371/journal.pone.0327209)
Supplement: S2 Fig — (DOCX) [file pone.0327209.s009.docx]

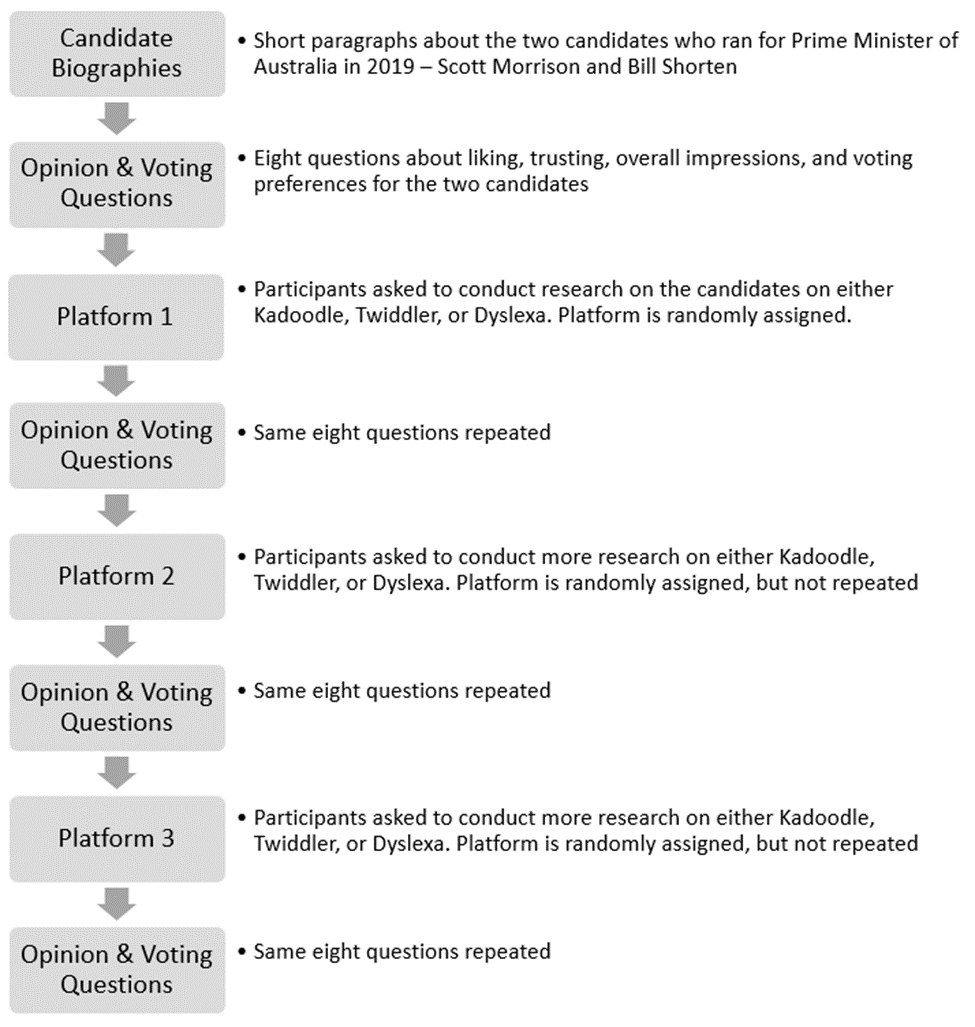


**S2 Fig. MPE Procedure**. Note that the three platforms were shown to each individual in a random order.
