# Supplementary material for: The Multiple Platforms Effect (MPE): A quantification of how exposure to similarly biased content on multiple online platforms might impact users
Source: PLoS One. 2025 Aug 1;20(8):e0327209. doi: 10.1371/journal.pone.0327209 (PMC12316238; doi:10.1371/journal.pone.0327209)
Supplement: S1 Table — (DOCX) [file pone.0327209.s012.docx]

**S1 Table. Demographic analysis by age.**

| **Platform** |  | ***n*** | **VMP (%)** |
| --- | --- | --- | --- |
| **1** | **< 36** | 176 | 41.9 |
|  | **≥ 36** | 189 | 42.9 |
|  | **Difference** | - | - 1.0 |
|  | **Statistic** | - | *z* = -0.19 |
|  | ***p*** | - | .85 NS |
| **2** | **< 36** | 176 | 48.8 |
|  | **≥ 36** | 189 | 63.7 |
|  | **Difference** | - | - 14.9 |
|  | **Statistic** | - | *z* = -2.87 |
|  | ***p*** | - | .004 |
| **3** | **< 36** | 176 | 65.1 |
|  | **≥ 36** | 189 | 68.1 |
|  | **Difference** | - | - 3.0 |
|  | **Statistic** | - | *z* = -0.61 |
|  | ***p*** | - | .54 NS |
