# Supplementary material for: The Multiple Platforms Effect (MPE): A quantification of how exposure to similarly biased content on multiple online platforms might impact users
Source: PLoS One. 2025 Aug 1;20(8):e0327209. doi: 10.1371/journal.pone.0327209 (PMC12316238; doi:10.1371/journal.pone.0327209)
Supplement: S2 Table — (DOCX) [file pone.0327209.s013.docx]

**S2 Table. Demographic analysis by gender.**

| **Platform** |  | ***n*** | **VMP (%)** |
| --- | --- | --- | --- |
| **1** | **Female** | 216 | 36.0 |
|  | **Male** | 149 | 54.0 |
|  | **Difference** | - | - 18.0 |
|  | **Statistic** | - | *z* = -3.41 |
|  | ***p*** | - | < .001 |
| **2** | **Female** | 216 | 52.6 |
|  | **Male** | 149 | 63.5 |
|  | **Difference** | - | - 10.9 |
|  | **Statistic** | - | *z* = -2.07 |
|  | ***p*** | - | .04 |
| **3** | **Female** | 216 | 59.6 |
|  | **Male** | 149 | 79.4 |
|  | **Difference** | - | - 19.8 |
|  | **Statistic** | - | *z* = -3.98 |
|  | ***p*** | - | < .001 |
