# Supplementary material for: The Multiple Platforms Effect (MPE): A quantification of how exposure to similarly biased content on multiple online platforms might impact users
Source: PLoS One. 2025 Aug 1;20(8):e0327209. doi: 10.1371/journal.pone.0327209 (PMC12316238; doi:10.1371/journal.pone.0327209)
Supplement: S3 Table — (DOCX) [file pone.0327209.s014.docx]

**S3 Table. Demographic analysis by education level.**

| **Platform** | **Level** | ***n*** | **VMP (%)** |
| --- | --- | --- | --- |
| **1** | **< Bachelors** | 145 | 34.6 |
|  | **≥ Bachelors** | 220 | 48.5 |
|  | **Difference** | - | - 13.9 |
|  | **Statistic** | - | *z* = -2.62 |
|  | ***p*** | - | .009 |
| **2** | **< Bachelors** | 145 | 39.7 |
|  | **≥ Bachelors** | 220 | 69.7 |
|  | **Difference** | - | - 30.0 |
|  | **Statistic** | - | *z* = -5.68 |
|  | ***p*** | - | < .001 |
| **3** | **< Bachelors** | 145 | 48.7 |
|  | **≥ Bachelors** | 220 | 80.8 |
|  | **Difference** | - | - 32.1 |
|  | **Statistic** | - | *z* = -6.44 |
|  | ***p*** | - | < .001 |
