# Supplementary material for: The Multiple Platforms Effect (MPE): A quantification of how exposure to similarly biased content on multiple online platforms might impact users
Source: PLoS One. 2025 Aug 1;20(8):e0327209. doi: 10.1371/journal.pone.0327209 (PMC12316238; doi:10.1371/journal.pone.0327209)
Supplement: S4 Table — (DOCX) [file pone.0327209.s015.docx]

**S4 Table. Demographic analysis by race/ethnicity.**

| **Platform** |  | ***N*** | **VMP (%)** |
| --- | --- | --- | --- |
| **1** | **White** | 271 | 45.7 |
|  | **Non-White** | 94 | 34.0 |
|  | **Difference** | - | + 11.7 |
|  | **Statistic** | - | *z* = 1.98 |
|  | ***p*** | - | .05 |
| **2** | **White** | 271 | 65.4 |
|  | **Non-White** | 94 | 34.0 |
|  | **Difference** | - | + 31.4 |
|  | **Statistic** | - | *z* = 5.30 |
|  | ***p*** | - | < .001 |
| **3** | **White** | 271 | 71.7 |
|  | **Non-White** | 94 | 54.0 |
|  | **Difference** | - | + 17.7 |
|  | **Statistic** | - | *z* = 3.15 |
|  | ***p*** | - | .002 |
