# Supplementary material for: The Multiple Platforms Effect (MPE): A quantification of how exposure to similarly biased content on multiple online platforms might impact users
Source: PLoS One. 2025 Aug 1;20(8):e0327209. doi: 10.1371/journal.pone.0327209 (PMC12316238; doi:10.1371/journal.pone.0327209)
Supplement: S5 Table — (DOCX) [file pone.0327209.s016.docx]

**S5 Table. Demographic analysis by political affiliation.**

| **Platform** |  | ***N*** | **VMP (%)** |
| --- | --- | --- | --- |
| **1** | **Liberal (left)** | 141 | 38.0 |
|  | **Conservative (right)** | 66 | 14.7 |
|  | **Difference** | - | + 23.3 |
|  | **Statistic** | - | *z* = 3.39 |
|  | ***p*** | - | < .001 |
| **2** | **Liberal (left)** | 141 | 53.5 |
|  | **Conservative (right)** | 66 | 26.5 |
|  | **Difference** | - | + 27.0 |
|  | **Statistic** | - | *z* = 3.64 |
|  | ***p*** | - | < .001 |
| **3** | **Liberal (left)** | 141 | 62.0 |
|  | **Conservative (right)** | 66 | 35.3 |
|  | **Difference** | - | + 26.7 |
|  | **Statistic** | - | *z* = 3.59 |
|  | ***p*** | - | < .001 |
