# Supplementary material for: The Multiple Platforms Effect (MPE): A quantification of how exposure to similarly biased content on multiple online platforms might impact users
Source: PLoS One. 2025 Aug 1;20(8):e0327209. doi: 10.1371/journal.pone.0327209 (PMC12316238; doi:10.1371/journal.pone.0327209)
Supplement: S6 Table — (DOCX) [file pone.0327209.s017.docx]

**S6 Table. Pre-exposure voting preferences measured on an 11-point scale, split by bias group (such that a negative value indicates preference for Scott Morrison and a positive value indicates preference for Bill Shorten).**

|  | **Pro-Scott Morrison** | **Pro-Bill Shorten** | **Control** | ***H*** | ***p*** |
| --- | --- | --- | --- | --- | --- |
| **Pre-Exposure Mean Voting Preference (SD)** | 0.21 (2.81) | -0.20 (2.61) | -0.27 (2.50) | 3.62 | .16 NS |
