# Supplementary material for: The Multiple Platforms Effect (MPE): A quantification of how exposure to similarly biased content on multiple online platforms might impact users
Source: PLoS One. 2025 Aug 1;20(8):e0327209. doi: 10.1371/journal.pone.0327209 (PMC12316238; doi:10.1371/journal.pone.0327209)
Supplement: S7 Table — (DOCX) [file pone.0327209.s018.docx]

**S7 Table. Changes in voting preferences measured on an 11-point scale, control group only (such that a negative value indicates preference for Scott Morrison and a positive value indicates preference for Bill Shorten).**

|  | **Pre-Exposure Mean Voting Preference (SD)** | **Post-Exposure Mean Voting Preference (SD)** | **Mean Difference** ^†^ | ***z*^‡^** | ***p*** |
| --- | --- | --- | --- | --- | --- |
| **Platform 1** | -0.27 (2.50) | -0.16 (2.72) | 0.11 | -0.55 | .58 NS |
| **Platform 2** | - | 0.04 (2.82) | 0.31 | -1.50 | .13 NS |
| **Platform 3** | - | -0.04 (2.85) | 0.23 | -1.18 | .24 NS |

*Note*: The means from 2nd exposure and 3rd exposure are being compared to the pre-exposure mean.

^†^The absolute value of the mean difference is shown.

**^‡^**The z values come from a Wilcoxon signed ranks test between post-exposure and pre-exposure ratings.
