# Supplementary material for: The Multiple Platforms Effect (MPE): A quantification of how exposure to similarly biased content on multiple online platforms might impact users
Source: PLoS One. 2025 Aug 1;20(8):e0327209. doi: 10.1371/journal.pone.0327209 (PMC12316238; doi:10.1371/journal.pone.0327209)
Supplement: S8 Table — (DOCX) [file pone.0327209.s019.docx]

**S8 Table. Pre-exposure opinion ratings of Bill Shorten and Scott Morrison measured on a 10-point scale, split by bias group.**

|  |  | **Pro-Scott Morrison Group Mean Rating (SD)** | **Pro-Bill Shorten Group Mean Rating (SD)** | **Control Group Mean Rating (SD)** | ***H*** | ***p*** |
| --- | --- | --- | --- | --- | --- | --- |
| **Scott Morrison** | **Impression** | 7.16 (1.80) | 7.20 (1.80) | 7.28 (1.60) | 0.35 | .84 NS |
|  | **Likeability** | 7.23 (1.85) | 7.13 (1.89) | 7.33 (1.57) | 0.26 | .88 NS |
|  | **Trust** | 6.15 (1.98) | 6.04 (1.94) | 6.30 (1.77) | 1.76 | .42 NS |
| **Bill Shorten** | **Impression** | 7.11 (1.84) | 7.05 (1.95) | 7.08 (1.82) | 0.06 | .97 NS |
|  | **Likeability** | 6.95 (1.80) | 6.71 (1.98) | 6.81 (1.82) | 0.81 | .67 NS |
|  | **Trust** | 6.15 (2.08) | 5.80 (2.04) | 6.15 (1.99) | 2.48 | .29 NS |
