# Supplementary material for: The Multiple Platforms Effect (MPE): A quantification of how exposure to similarly biased content on multiple online platforms might impact users
Source: PLoS One. 2025 Aug 1;20(8):e0327209. doi: 10.1371/journal.pone.0327209 (PMC12316238; doi:10.1371/journal.pone.0327209)
Supplement: S9 Table — (DOCX) [file pone.0327209.s020.docx]

**S9 Table. Pre- and post-exposure opinion ratings of Scott Morrison and Bill Shorten measured on a 10-point scale, control group only.**

|  | |  | **Scott Morrison Mean (SD)** |  | | **Bill Shorten Mean (SD)** |  | | |  |  |
| --- | --- | --- | --- | --- | --- | --- | --- | --- | --- | --- | --- |
|  |  | | **Pre** | **Post** | **Diff** | **Pre** | **Post** | **Diff** | ***z***^†^ | | ***p*** |
| **Platform 1** | **Impression** | | 7.28 (1.60) | 6.68 (1.83) | - 0.60 | 7.08 (1.81) | 6.58 (1.99) | - 0.50 | -0.44 | | .66 NS |
|  | **Likeability** | | 7.33 (1.57) | 6.72 (1.93) | - 0.61 | 6.81 (1.82) | 6.43 (1.91) | - 0.38 | -1.08 | | .28 NS |
|  | **Trust** | | 6.30 (1.77) | 6.15 (1.99) | - 0.15 | 6.15 (1.99) | 5.96 (2.09) | - 0.19 | -1.13 | | .26 NS |
| **Platform 2** | **Impression** | | - | 6.44 (1.99) | - 0.84 | - | 6.26 (2.13) | - 0.82 | -0.19 | | .85 NS |
|  | **Likeability** | | - | 6.37 (1.99) | - 0.96 | - | 6.15 (2.11) | - 0.66 | -1.59 | | .11 NS |
|  | **Trust** | | - | 6.04 (2.11) | - 0.26 | - | 5.83 (2.19) | - 0.32 | -0.31 | | .76 NS |
| **Platform 3** | **Impression** | | - | 6.39 (1.89) | - 0.89 | - | 6.13 (2.02) | - 0.95 | -0.16 | | .87 NS |
|  | **Likeability** | | - | 6.26 (1.94) | - 1.07 | - | 6.05 (2.03) | - 0.76 | -1.67 | | .10 NS |
|  | **Trust** | | - | 5.98 (2.11) | - 0.32 | - | 5.73 (2.17) | - 0.42 | -0.43 | | .67 NS |

*Note*: The means from 2nd exposure and 3rd exposure are being compared to the pre-exposure mean.

^†^The z values come from Wilcoxon signed ranks test between post-exposure minus pre-exposure ratings for Scott Morrison and the post-exposure minus pre-exposure ratings for Bill Shorten.
